# From Inflammation to Malignancy: Diagnosing Ocular Adnexal Diffuse Large B-Cell Lymphoma Beyond Dacryocystitis

**DOI:** 10.7759/cureus.107437

**Published:** 2026-04-21

**Authors:** Muhammad Yusuf Abdurrahman, Khairul-Anwar Ibrahim, Raja Nor Farahiyah Raja Othman, Akmal Haliza Zamli, Khairidzan Mohd. Kamal

**Affiliations:** 1 Ophthalmology, International Islamic University Malaysia, Kuala Lumpur, MYS; 2 Ophthalmology, International Islamic University Malaysia Medical Specialist Center (IMSC), Kuantan, MYS; 3 Ophthalmology, Kulliyyah of Medicine, International Islamic University Malaysia, Kuantan, MYS; 4 Ophthalmology, Sultan Ahmad Shah Medical Center, International Islamic University Malaysia, Kuantan, MYS; 5 Ophthalmology, Hospital Serdang, Serdang, MYS; 6 Ophthalmology, Hospital Sultanah Nur Zahirah, Kuala Terengganu, MYS; 7 Ophthalmology, Hospital Tengku Ampuan Afzan, Kuantan, MYS

**Keywords:** allergic rhinitis, chronic dacryocystitis, extranodal marginal zone lymphoma, facial swelling, fine needle aspiration cytology, nasolacrimal duct obstruction, ocular adnexal lymphoma, periocular swelling, proptosis, submandibular lymphadenopathy

## Abstract

This case report discusses a 16-year-old male patient with a history of recurrent allergic rhinitis and sinusitis, who initially presented with left eyelid swelling, proptosis, and progressive facial edema extending from the left medial canthus to the nasal bridge. He was treated for nearly a year under the working diagnosis of chronic left dacryocystitis with multiple courses of topical, oral, and intravenous antibiotics. However, the patient developed worsening proptosis, progressive left-sided vision loss, and left submandibular lymphadenopathy, which ultimately guided the diagnostic focus toward ocular adnexal lymphoma (OAL) involving the left orbit.

Despite multiple imaging studies and histopathological assessments, it took nearly a year to identify the exact pathology. This case report details the diagnostic journey, treatment strategies, and interventions employed to preserve the patient’s vision, ultimately enabling him to resume normal activities.

We emphasize that in cases of chronic, unresponsive dacryocystitis, ocular adnexal lymphoma should be considered in the differential diagnosis. Early surgical intervention plays a critical role in optimizing visual outcomes and should be pursued to avoid further morbidity associated with delayed diagnosis.

## Introduction

Ocular adnexal lymphoma represents a heterogeneous group of extranodal lymphomas involving the orbit, eyelids, lacrimal gland, and conjunctiva. It accounts for approximately 1-2% of all non-Hodgkin lymphomas and is the most common primary orbital malignancy, comprising up to 55% of orbital tumors in large clinicopathologic series [1-2].

Diffuse large B-cell lymphoma (DLBCL) is an aggressive subtype of non-Hodgkin lymphoma and one of the more common high-grade lymphomas affecting the orbit. It typically presents as a rapidly enlarging orbital mass with associated compressive symptoms. However, in its early stages, orbital lymphoma may mimic benign or inflammatory conditions, including dacryocystitis, leading to potential delays in diagnosis and management.

This diagnostic challenge is particularly relevant in younger patients, where orbital lymphoma is exceedingly rare and often not considered in the initial differential diagnosis. Features such as persistent or progressive swelling despite appropriate antibiotic therapy, atypical location or extension of inflammation, firm non-tender masses, and associated orbital or facial involvement should raise suspicion for an underlying malignant process rather than infection.

Early recognition and prompt histopathological confirmation are critical, as DLBCL requires timely systemic therapy and multidisciplinary management to optimize outcomes. Although imaging plays a key role in identifying orbital involvement, definitive diagnosis relies on tissue biopsy and immunohistochemical analysis.

In this report, we describe a rare case of orbital DLBCL in a 16-year-old Malay male patient who presented with a four-month history of progressive unilateral eyelid swelling, epiphora, and facial swelling. The patient was initially treated for presumed infection with partial symptomatic improvement, followed by clinical deterioration that prompted further investigation. This case highlights the importance of recognizing red-flag features that distinguish malignancy from infection and emphasizes the need for early multidisciplinary evaluation in atypical or non-resolving orbital presentations.

## Case presentation

A 16-year-old Malay male patient with a background history of allergic rhinitis (which was perennial), presented to the ophthalmology clinic with a four-month history of progressive left medial eyelid swelling associated with epiphora and intermittent seropurulent discharge. The swelling initially began in September 2023 and was painless. He had previously sought treatment at a private clinic, where oral antibiotics led to partial resolution of associated facial swelling; however, the periocular swelling persisted and gradually enlarged.

**Figure 1:**
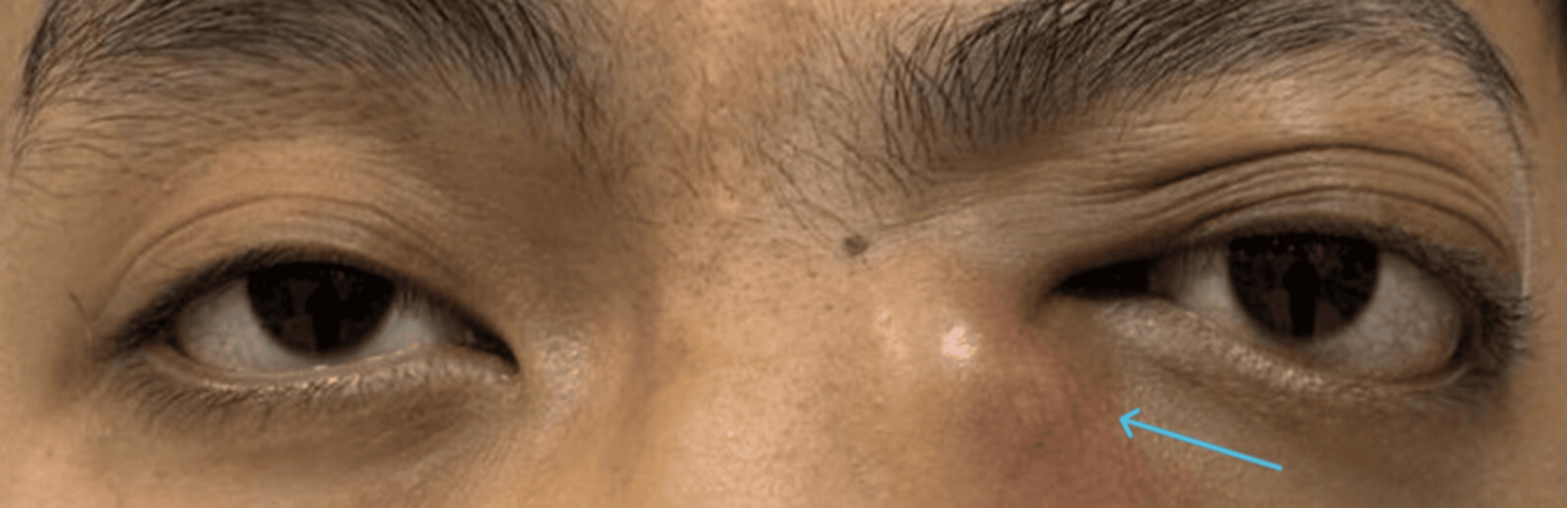
One day before debulking of orbital mass, swollen inferior left medial canthus, with left proptosis.

On initial ophthalmic evaluation in January 2024, best-corrected visual acuity was 6/9 in both eyes. There was no relative afferent pupillary defect. During this time, there was no obvious proptosis. Hertel exophthalmometry was 16 mm for both eyes. Examination revealed a localized, non-tender swelling over the left medial canthus extending toward the nasal bridge. Digital pressure over the swelling expressed seropurulent discharge from the superior punctum. The overlying skin was non-erythematous and non-warm. Anterior segment examination was otherwise unremarkable, intraocular pressures were within normal limits, and fundus examination showed no optic disc edema. Extraocular movements were full at this stage, and there was no significant proptosis. Figure 1 shows the left orbital region before the operation.

**Figure 2:**
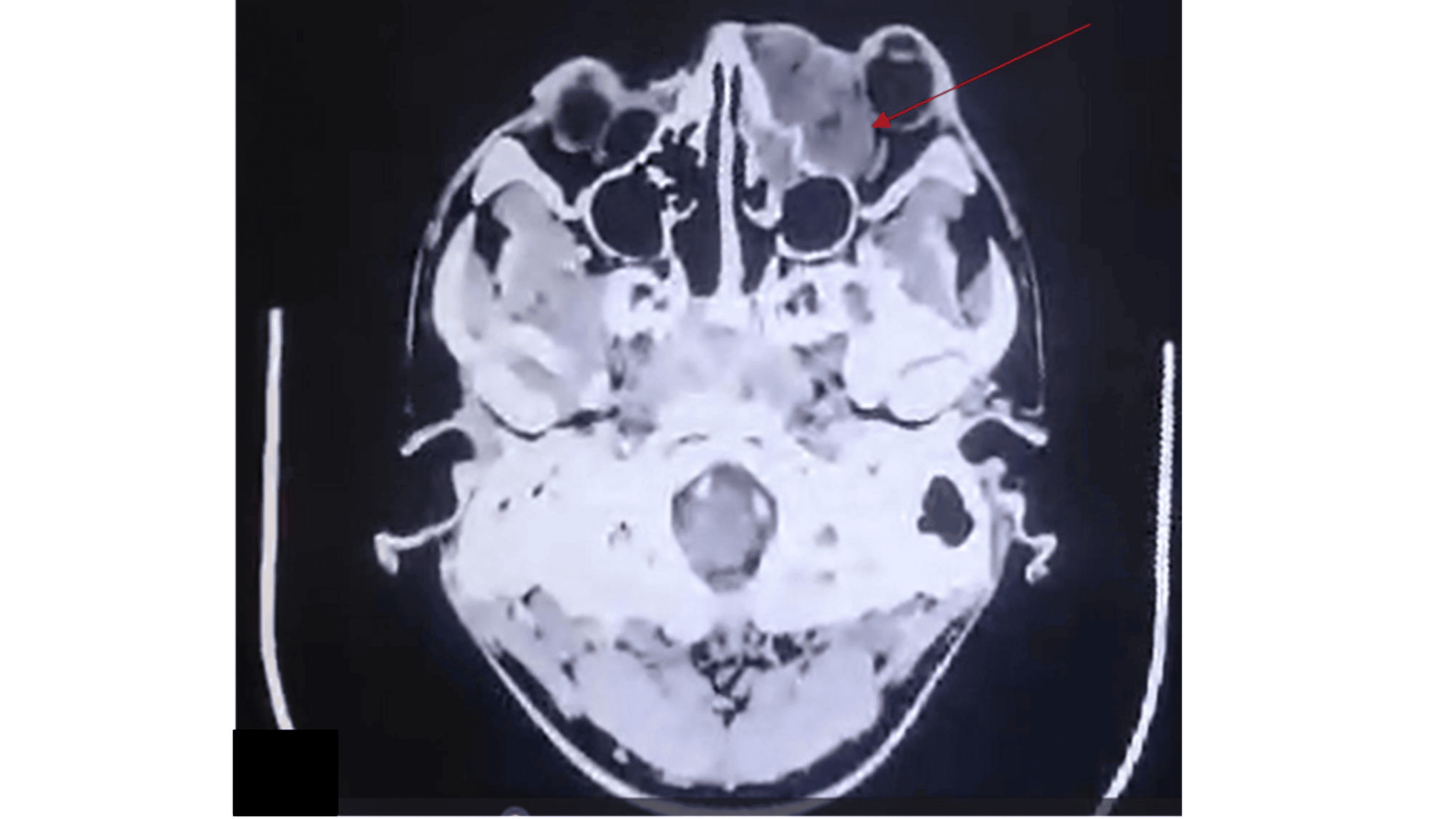
Enhancing extraconal mass in the left medial orbit, displacing the left globe and left optic nerve temporally.

Computed tomography (CT) of the orbits and paranasal sinuses, as shown in Figure 2, demonstrated a heterogeneously enhancing extraconal mass in the left medial orbit measuring approximately 4.2 × 3.2 × 3.8 cm. The lesion caused thinning of the inferomedial orbital wall, displaced the medial and inferior rectus muscles, and indented the globe without evidence of optic nerve involvement. Medially, the mass extended into the left ethmoidal sinus and nasolacrimal duct, causing ductal expansion and bony remodeling. Based on these findings, an initial working diagnosis of chronic dacryocystitis with an associated medial orbital mass was considered.

By late February 2024, the patient developed progressive proptosis, mild left hypertropia, and restricted abduction of the left eye. Hertel exophthalmometry revealed 21 mm in the left eye compared to 16 mm in the right. In addition, a palpable left submandibular lymph node was noted, localized, measuring around 1.5 cm x 1 cm, which had a smooth surface, was firm in consistency, and was non-tender. Fine needle aspiration cytology of the lymph node demonstrated reactive lymphoid hyperplasia consistent with acute lymphadenitis, with no malignant cells identified. The patient was admitted for intravenous antibiotics and scheduled for an elective left anterior orbitotomy with excision biopsy.

However, in early March 2024, he re-presented with acute worsening of proptosis and a reduction in left eye visual acuity to 6/24. A grade 1 relative afferent pupillary defect was present, raising concern for compressive optic neuropathy. Repeat CT imaging showed interval enlargement of the medial orbital mass with new compression and displacement of the left optic nerve, prompting urgent surgical intervention.

The patient subsequently underwent a combined left medial orbitotomy with excision biopsy and endoscopic tumor debulking in collaboration with the otorhinolaryngology team. Intraoperatively, a friable whitish mass was identified involving the medial orbit, anterior ethmoid sinus, and maxillary sinus, with external compression of the lacrimal sac but no intraluminal tumor involvement.

**Figure 3:**
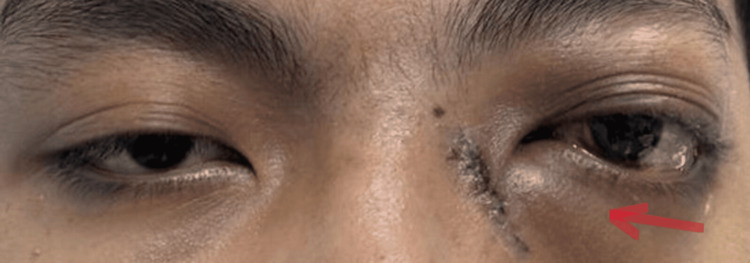
One week post debulking of left orbital mass, left proptosis has reduced.

Figure 3 shows one week post-debulking of the left orbital mass, which revealed a marked reduction in the left eye proptosis.

Histopathological examination of the excised tissue revealed sheets of malignant lymphoid cells composed of medium to large atypical cells with vesicular nuclei, prominent nucleoli, and frequent mitotic figures. These findings were diagnostic of diffuse large B-cell lymphoma. However, as this case report is retrospective in nature, the histopathology slides could not be retrieved from archival storage and, therefore, are not presented in this report.

The patient was subsequently referred to the hematology-oncology team for systemic evaluation and chemotherapy. At one-month follow-up, his left eye best-corrected visual acuity had improved to 6/9, the relative afferent pupillary defect had resolved, proptosis had reduced, and extraocular motility showed significant improvement. He remains under ongoing oncologic and ophthalmologic follow-up.

## Discussion

Dacryocystitis is an infection or inflammation of the lacrimal sac, typically characterized by symptoms such as redness, tenderness, swelling over the sac area, and epiphora (Fatani & AlSuhaibani, 2024) [3]. The patient in this case presented with recurrent episodes exhibiting the clinical features of dacryocystitis, which initially responded to antibiotic therapy. However, persistent swelling despite multiple treatments suggested that the underlying pathology was not fully addressed by conventional management. Furthermore, the presence of long-standing allergic rhinitis in this patient likely contributed to predisposing factors, as nasal congestion and impaired drainage can facilitate the development of such infections (Tugrul et al., 2021) [4].

Ocular adnexal lymphoma typically presents with symptoms such as proptosis, palpable mass, swelling, ptosis, restricted eye movement, eye displacement, and diplopia (Chung & Son, 2022) [5]. In this case, diagnostic reconsideration was prompted when the patient re-presented with features suggestive of orbital involvement, including reduced visual acuity in the left eye, a positive relative afferent pupillary defect (RAPD), and progressive proptosis. These findings are not characteristic of uncomplicated dacryocystitis and raised suspicion for an underlying orbital mass, leading to further diagnostic work-up and eventual diagnosis of orbital lymphoma.

Although ocular adnexal lymphoma is rarely reported to mimic dacryocystitis, this may occur when tumor-related compression of the lacrimal sac or nasolacrimal duct results in secondary obstruction and infection (Mechel et al., 2022) [6]. This overlap can obscure the underlying diagnosis, particularly in the early stages when inflammatory features predominate. In the present case, the initial management with antimicrobial therapy was appropriate based on the presenting features. However, the persistence of symptoms and the subsequent development of optic nerve involvement highlighted the need for further evaluation. Intraoperative findings confirmed an orbital mass compressing the lacrimal sac, leading to nasolacrimal duct obstruction and secondary dacryocystitis. This underscores the importance of considering ocular adnexal lymphoma in the differential diagnosis, particularly in cases that are atypical or resistant to standard treatment (Ponzoni et al., 2021) [7].

This case illustrates the diagnostic challenge in distinguishing common benign conditions from underlying malignancy. While dacryocystitis is frequently encountered and often managed conservatively, this case demonstrates that persistent or atypical presentations warrant closer scrutiny. Importantly, the key clinical lesson from this case is not the need for early surgical intervention in all cases, but rather the importance of timely escalation of investigation when red flag features are present.

Specifically, persistent dacryocystitis-like symptoms, particularly when accompanied by visual impairment, proptosis, ophthalmoplegia, or RAPD, should prompt early orbital imaging and consideration of tissue diagnosis. In this patient, CT imaging was crucial in identifying the extraconal mass and its relationship to adjacent structures, thereby guiding further management. Additional evaluation, including fine needle aspiration cytology of an enlarged submandibular lymph node, provided further evidence supporting the diagnosis of lymphoma (Stephen et al., 2021) [8].

The diagnosis of orbital lymphoma requires a high index of suspicion, particularly when symptoms are atypical or fail to resolve with initial treatment (Olsen & Heegaard, 2019) [9]. The overlap of symptoms between benign and malignant conditions underscores the need for maintaining a broad differential diagnosis, especially when the clinical course deviates from expected recovery. Early detection of orbital lymphoma is crucial, as delayed recognition may lead to progressive disease and potential visual loss.

## Conclusions

In conclusion, this case supports the observation that orbital lymphoma can mimic dacryocystitis and highlights the risk of delayed diagnosis. However, the findings from this single case more appropriately emphasize the need for clinical vigilance, with early imaging and biopsy in persistent or atypical presentations, rather than broad recommendations for early surgical intervention.

We suggest that in any case of recurrent, unresolved dacryocystitis, despite classic treatment - especially with optic nerve involvement, proptosis, and lymphadenopathy - ocular adnexal lymphoma should be excluded early, and early orbital imaging is beneficial to aid further management and improve the patient's visual outcome. By promptly ruling out orbital lymphoma, treatment can be tailored more effectively, improving patient outcomes and preventing unnecessary complications.
